# Mettl3-mediated m^6^A RNA methylation regulates the fate of bone marrow mesenchymal stem cells and osteoporosis

**DOI:** 10.1038/s41467-018-06898-4

**Published:** 2018-11-14

**Authors:** Yunshu Wu, Liang Xie, Mengyuan Wang, Qiuchan Xiong, Yuchen Guo, Yu Liang, Jing Li, Rui Sheng, Peng Deng, Yuan Wang, Rixin Zheng, Yizhou Jiang, Ling Ye, Qianming Chen, Xuedong Zhou, Shuibin Lin, Quan Yuan

**Affiliations:** 1 State Key Laboratory of Oral Diseases & National Clinical Research Center for Oral Diseases, West China Hospital of Stomatology, Sichuan University, 610041 Chengdu, China; 2 Center for Translational Medicine, The First Affiliated Hospital, Sun Yat-sen University, Guangzhou, 510080 China; 3 Institute for Advanced Study, Shenzhen University, Shenzhen, 518060 China

## Abstract

N^6^-methyladenosine (m^6^A) is the most abundant epigenetic modification in eukaryotic mRNAs and is essential for multiple RNA processing events during mammalian development and disease control. Here we show that conditional knockout of the m^6^A methyltransferase *Mettl3* in bone marrow mesenchymal stem cells (MSCs) induces pathological features of osteoporosis in mice. *Mettl3* loss-of-function results in impaired bone formation, incompetent osteogenic differentiation potential and increased marrow adiposity. Moreover, *Mettl3* overexpression in MSCs protects the mice from estrogen deficiency-induced osteoporosis. Mechanistically, we identify PTH (parathyroid hormone)/Pth1r (parathyroid hormone receptor-1) signaling axis as an important downstream pathway for m^6^A regulation in MSCs. Knockout of *Mettl3* reduces the translation efficiency of MSCs lineage allocator Pth1r, and disrupts the PTH-induced osteogenic and adipogenic responses in vivo. Our results demonstrate the pathological outcomes of m^6^A mis-regulation in MSCs and unveil novel epitranscriptomic mechanism in skeletal health and diseases.

## Abstract (web summary)

mRNA modifications have been shown to regulate mammalian development and disease. Here the authors show that the m^6^A methyltransferase Mettl3 ensures translational efficiency of the mesenchymal stem cell lineage allocator Pth1r, promoting osteogenesis and protecting from osteoporosis.

## Introduction

Bone marrow mesenchymal stem cells (MSCs) are the common progenitors for osteoblasts and marrow adipocytes. The reciprocal balance between osteogenic and adipogenic differentiation of MSCs is under tightly spatiotemporal controls to safeguard skeletal health^1–3^. Aged-related osteoporosis is featured with low bone mass and excessive accumulation of adipose tissue in bone marrow milieu^1–3^. Under aging or other pathological stimuli such as hormone disorders, the bone marrow MSCs undergo preferential shift of differentiation towards adipocytes, resulting in the increase in marrow adiposity and progressive bone loss^1,4,5^. These alterations in bone micro-architecture lead to the increased skeletal fragility and susceptibility to fracture. However, the explicit mechanisms under which the lineage allocation of MSCs favors adipogenic to osteogenic lineage remain unclear.

N^6^-methyladenosine (m^6^A) is the most prevalent post-transcriptional internal mRNA modification that regulates the fine-tuning of a variety of biological processes^6–8^. In mammals, m^6^A is catalyzed by the methyltransferase complex consisting of an enzymatic subunit METTL3, a substrate recognition subunit METTL14 and a regulatory subunit WTAP^9–11^, and it can be erased by demethylases FTO and ALKBH5^12,13^. At molecular level, m^6^A marks are dynamically installed and removed from their regulated transcripts to adjust RNA metabolisms, including alternative splicing, RNA stability, and translation, in response to multiple signaling cues during normal cellular processes or under stress or diseases^14–19^. Alterations in expressions of m^6^A methylated transcripts subsequently affect the cellular function, identity and stemness of their residing cells^20–24^, determining cell fate in a context-dependent manner. Recent in vivo studies made great breakthroughs in deciphering the intriguing involvement of m^6^A in mammalian development. Aberrant m^6^A levels upon *Mettl3* and/or *Mettl14* deficiency attenuated cell cycle progression, and disrupted the proper lineage commitment and differentiation of functional stem cells, resulting in retarded neurogenesis, immune defects, and infertility^25–28^. Given the strong correlation of m^6^A with health and diseases, we focus on exploring the potential involvement of m^6^A in bone homeostasis, about which little was known.

By generating conditional knockout and knock-in mutant mice, we unveil a crucial effect of m^6^A functioning in modulating MSCs differentiation, and discover PTH/Pth1r signaling axis as an important m^6^A downstream mechanism pathway. Our findings provide a new insight in the pivotal regulatory role of m^6^A in skeletal health and diseases.

## Results

### Conditional deletion of *Mettl3* in MSCs leads to low bone mass and high marrow adiposity

To study the potential role of Mettl3 in bone marrow MSCs lineage allocation and bone diseases, we first examined the expression of Mettl3 in mouse bone tissues. Immunostaining showed that Mettl3 is prevalently expressed in the bone cells and bone marrow of mice, but absent in chondrocytes of the growth plate zone (Supplementary Fig. 1). Since genomic *Mettl3* knockout is embryonic lethal^20^, we generated *Mettl3* flox (*Mettl3*^*fl*/+^) mice using CRISPR/Cas9 technique (Supplementary Fig. 2a), and then bred *Mettl3*^*fl*/+^ mice with *Prx1-Cre* transgenic mice to obtain conditional homozygous *Mettl3* knockout mice, *Prx1-Cre;Mettl3*^*fl/fl*^ mice, as well as heterozygous *Mettl3* knockout mice, *Prx1-Cre;Mettl3*^*fl/+*^ mice (Supplementary Fig. 2b,c). *Prx1-Cre;Mettl3*^*fl/fl*^ mice were viable, though with a lower survival rate (Supplementary Fig. 2d), and exhibited a sex bias towards maleness (about 4.5:1), in consistent with previous studies^17,29^. They had smaller size, lighter weight and retarded growth features compared to their *Mettl3*^*fl/fl*^ control littermates at 4 weeks of age. Immunostaining of femur paraffin sections and western blot analysis proved the deletion of *Mettl3* was successful, which subsequently reduced the m^6^A level in MSCs (Supplementary Fig. 2e-g).

**Fig. 1:**
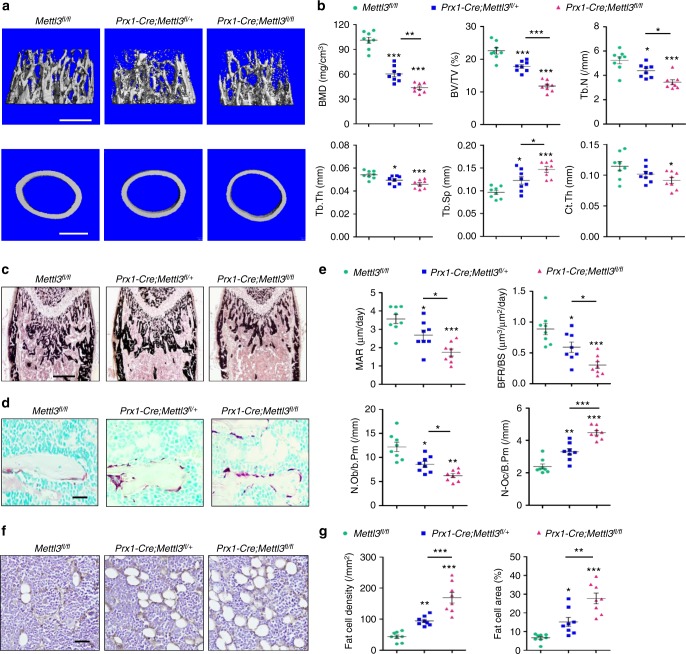
Deletion of *Mettl3* in MSCs leads to low bone mass and high marrow adiposity. **a** Representative μCT images of distal femurs and midshaft cortical bone. Scale bar, 500 μm. **b** Quantitative μCT analyses of distal end of femurs (*n* = 8). **c** Von Kossa staining of undecalcified sections of femurs. Scale bar, 500 μm. **d** TRAP staining of femur sections. Scale bar, 50 μm. **e** Histomorphometric analyses of trabecular bone from the femur metaphysic (*n* = 8). **f** Immunohistochemical staining of FABP4. Scale bar, 50 μm. **g** Number and area of adipocytes in the distal marrow per tissue area (*n* = 8). Data are expressed as mean ± s.e.m.; *P < 0.05, **P < 0.01, ***P < 0.001 by one-way ANOVA with Tukey’s post hoc test

Micro-computed tomography (μCT) analysis of trabecular bone of the distal femur metaphysis revealed that bone mineral density (BMD) and bone volume/tissue volume ratio (BV/TV) were significantly reduced in *Prx1-Cre;Mettl3*^*fl/fl*^ and *Prx1-Cre;Mettl3*^*fl/+*^ male mice compared to their *Mettl3*^*fl/fl*^ littermates (Fig. 1a, b). In addition, *Mettl3* deletion also diminished the trabecular number (Tb.N), trabecular thickness (Tb.Th) and midshaft cortical thickness (Ct.Th), while increasing the trabecular separation (Tb.Sp) (Fig. 1b). Von Kossa staining of the undecalcified sections further confirmed the low bone mass phenotype of *Prx1-Cre;Mettl3*^*fl/fl*^ mice (Fig. 1c). Moreover, the *Mettl3* conditional knockout mice had slower mineral apposition rate (MAR) and bone formation rate (BFR), with also less osteoblast number (N.Ob/B.Pm) (Fig. 1e), suggesting that deletion of *Mettl3* in MSCs contributed to the impaired bone formation in mice. We also observed a more active osteoclast state in *Prx1-Cre;Mettl3*^*fl/fl*^ mice (Fig. 1d, e), supporting a faster bone tissue breakdown occurring in the *Mettl3* conditional knockout mice. *Mettl3* knockout only led to a slightly yet not significantly shortened growth plate and hypertrophic zones (Supplementary Fig. 3).

Interestingly, we observed that the loss of bone mass is associated with apparently increased bone marrow adipose tissue (MAT) accumulation in the *Prx1-Cre;Mettl3*^*fl/fl*^ mice (Fig. 1f). Both the number and the density of marrow adipocytes were elevated in *Prx1-Cre;Mettl3*^*fl/fl*^ mice compared to their *Mettl3*^*fl/fl*^ controls (Fig. 1g). Overall, the skeletal manifestations of *Mettl3* conditional knockout mice resemble the pathological phenotypes of osteoporosis, implying a lineage allocation disorder in MSCs with low m^6^A methylation level.

### Loss of *Mettl3* in MSCs leads to compromised osteogenic potential and increased adipogenic differentiation

**Fig. 2:**
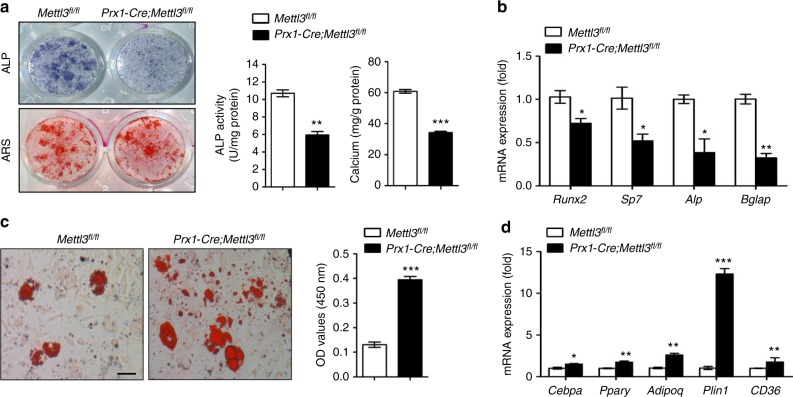
*Mettl3*-deficient MSCs exhibit compromised osteogenic potential and increased adipogenic differentiation. **a** Representative images and quantitative analyses of ALP and ARS staining of MSCs isolated from *Mettl3*^*fl/fl*^ and *Prx1-Cre;Mettl3*^*fl/fl*^ mice. **b** qRT-PCR analyses of the expression of *Runx2*, *Sp7*, *Alp* and *Bglap* under osteogenic condition. **c** Representative images and quantitative analysis of oil red O staining. Scale bar, 100 μm. **d** qRT-PCR analyses of the expression of *Cebpα*, *Pparγ*, *Adipoq, Plin1*, and *CD36* under adipogenic condition. Results are from three independent experiments. Data are expressed as mean ± s.e.m.; *P < 0.05, **P < 0.01, ***P < 0.001 by two-tailed Student’s *t* test

Next, we isolated MSCs from *Mettl3*^*fl/fl*^ and *Prx1-Cre;Mettl3*^*fl/fl*^ mice to verify their osteogenic and adipogenic potentials in vitro. The incompetent osteogenic differentiation ability of *Mettl3* deleted MSCs was evidenced by weaker alkaline phosphatase staining (ALP) activity and less calcium mineralization (Fig. 2a), and the downregulated expression of osteogenic markers, including *Runx2*, *Sp7*, *Alp*, and *Bglap* (Fig. 2b). On the contrary, the increased intensity of oil red O staining (Fig. 2c) and significantly elevated expression of adipogenic factors, including *Pparγ*, *Cebpα*, *Adipoq*, *Plin1*, and *CD36* (Fig. 2d), demonstrated the enhanced adipogenic potential of *Prx1-Cre;Mettl3*^*fl/fl*^ MSCs.

### Deletion of *Mettl3* in ***Lepr***^+^ MSCs results in bone impairment and marrow fat accumulation

**Fig. 3:**
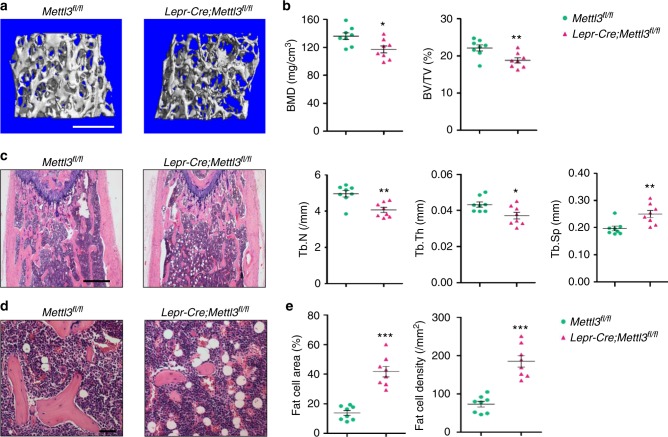
Deletion of *Mettl3* in *Lepr*^*+*^ MSCs results in bone impairment and marrow fat accumulation. **a** Representative μCT images of distal femurs of 4-month-old male mice. Scale bar, 500 μm. **b** Quantitative μCT analyses of distal end of femurs (*n* = 8). **c** H&E staining of femoral sections from *Mettl3*^*fl/fl*^ and *Lepr-Cre;Mettl3*^*fl/fl*^ male mice. Scale bar, 500 μm. **d** Representative H&E staining of femur sections exhibiting the marrow adipose tissue. Scale bar, 50 μm. **e** Number and area of adipocytes in the distal marrow per tissue area (*n* = 8). Data are expressed as mean ± s.e.m.; *P < 0.05, **P < 0.01, ***P < 0.001 by two-tailed Student’s *t* test

We next sought to examine whether Mettl3 has comparable importance in a later stage of bone formation. To this end, we used *Lepr-Cre* line, which mainly targets at MSCs that majorly form bone tissues in adult bone marrow^30,31^. *Lepr-Cre;Mettl3*^*fl/fl*^ mice recapitulated osteogenic block and adipogenic promotion seen in *Prx1-Cre;Mettl3*^*fl/fl*^ mice. Deletion of *Mettl3* in *Lepr*^*+*^ MSCs led to less trabecular bone in distal femoral, with a decrease in the number and thickness of trabeculae, as presented by lower BMD, BV/TV, Tb.N, and Tb.Th (Fig. 3a, b). Histological examination revealed an increased abundance of adipose tissue in bone marrow milieu of *Lepr-Cre;Mettl3*^*fl/fl*^ mice in contrast to their *Mettl3*^*fl/fl*^ littermates (Fig. 3c, d). Quantitative analysis of fat cells showed that both the fat cell number and density of the mutants increased more than doubled that of the controls (Fig. 3e).

### Overexpression of ***Mettl3*** prevents estrogen deficiency-induced osteoporosis

**Fig. 4:**
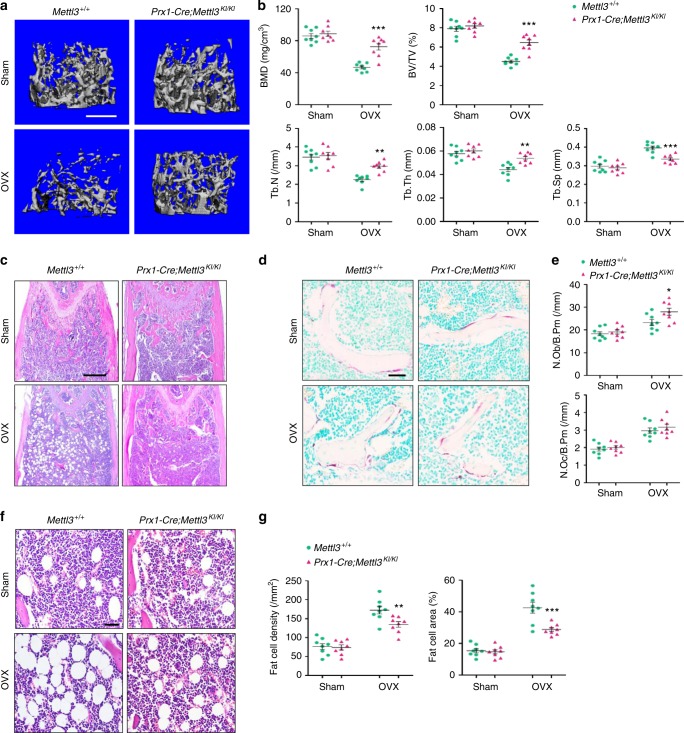
Overexpression of *Mettl3* in MSCs prevents estrogen deficiency-induced osteoporosis. **a** Representative images of μCT reconstruction of distal femurs. Scale bar, 500 μm. **b** Quantitative μCT analyses of metaphysis region of distal femurs (*n* = 8). **c** H&E staining of femoral sections from *Mettl3*^*+l+*^ and *Prx1-Cre;Mettl3*^*KI/KI*^ female mice following sham and OVX. Scale bar, 500 μm. **d** TRAP staining of femur sections. Scale bar, 50 μm. **e** Histomorphometric analyses of distal femurs following sham and OVX (*n* = 8). **f** Representative H&E staining of femur sections exhibiting the marrow adipose tissue. Scale bar, 50 μm. **g** Number and area of adipocytes in the distal marrow per tissue area (*n* = 8). Data are expressed as mean ± s.e.m.; *P < 0.05, **P < 0.01, ***P < 0.001 by two-way ANOVA with Bonferroni post tests

Considering the pathologic effect of *Mettl3* deletion in triggering osteoporosis, we questioned whether *Mettl3* overexpression is capable to enhance skeletal health or prevent bone disorders. To this end, we generated the *Prx1-Cre* driven *Mettl3-tdTomato* knock-in mice (*Prx1-Cre;Mettl3*^*KI/KI*^) to conditionally overexpress *Mettl3* in MSCs (Supplementary Fig. 4a, b). *Prx1-Cre;Mettl3*^*KI/KI*^ mice were viable and born at an expected Mendelian ratio. To study the potential protective role of *Mettl3* overexpression in osteoporosis, 9-week-old *Prx1-Cre;Mettl3*^*KI/KI*^ female mice and their control littermates were subjected to ovariectomy (OVX), a classic model to induce the postmenopausal osteoporosis^32^, in which the estrogen deprivation upon postmenopause gave rise to imbalanced bone remodeling and increased marrow fat deposition^33^. Although *Prx1-Cre;Mettl3*^*KI/KI*^ mice did not exhibit significant change of bone mass in normal state, we observed a less decrease of trabecular bone density and bone volume in *Mettl3* knock-in mice after OVX, compared to their controls (Fig. 4a, b). The decline in Tb.N and Tb.Th, as well as the increase in Tb.Sp were less obvious when *Mettl3* was overexpressed (Fig. 4b). In addition, *Mettl3* overexpression elevated osteoblast numbers in mice following OVX without affecting the osteoclast activity (Fig. 4c-e). H&E staining and histomorphology analysis revealed an excessive accumulation of marrow adipocytes in ovariectomized control mice whilst the increase was minor in the ovariectomized *Prx1-Cre;Mettl3*^*KI/KI*^ mice (Fig. 4f, g).

### m^6^A regulates the translation of parathyroid hormone receptor-1

**Fig. 5:**
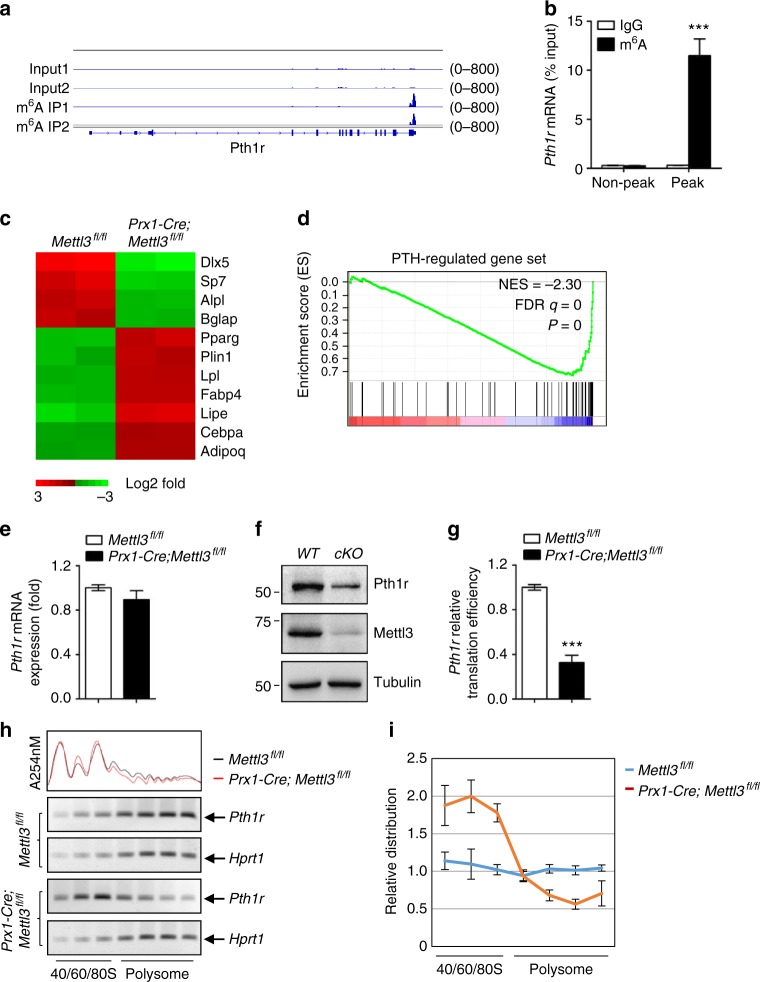
Mettl3-mediated m^6^A modification in MSCs regulates Pth1r translation. **a** m^6^A MeRIP-Seq revealed that *Pth1r* has high enriched and specific m^6^A peak near its translation stop codon. **b** MeRIP-qPCR validation of *Pth1r* m^6^A peak specificity. **c** Heatmap of representative osteogenesis and adipogenesis associated genes. **d** GSEA showed decreased enrichment of PTH-regulated genes in *Mettl3*-deficient MSCs. **e** qRT-PCR analysis of *Pth1r* expression. **f** Western blot analysis of Pth1r. *WT*: *Mettl3*^*fl/fl*^, *cKO*: *Prx1-Cre;Mettl3*^*fl/fl*^. **g** Translation efficiency of Pth1r. **h** PCR analysis of *Pth1r* mRNA in different polysome gradient fractions in the *Mettl3* knockout and control cells. Hprt1 was used as a control. **i** Quantification of *Pth1r* mRNA relative distribution in the *Mettl3* depleted and control cells. The band intensities in **g** were analyzed by Image J. The relative amount of *Pth1r* or *Hprt1* mRNA in each fraction was calculated as percentage of the total. Then the relative distribution of *Pth1r* mRNA was plotted by normalizing the percentage of *Pth1r* mRNA to *Hprt1* mRNA in each fraction. Results are from three independent experiments. Data are expressed as mean ± s.e.m.; *P < 0.05, **P < 0.01, ***P < 0.001 by two-tailed Student’s *t* test

To dissect the underlying mechanism of Mettl3 in regulation of MSCs fate and bone formation, we performed the m^6^A MeRIP-Seq to identify the critical m^6^A targets in MSCs (Supplementary Data 1). As shown in Supplementary Figure 5a-c, the “GGAC” sequence motif is enriched in the m^6^A sites identified in MSCs (Supplementary Fig. 5a), majority of the m^6^A sites are located in the CDS and the 3′UTR regions, with a small subset of peaks located in the 5′UTR and intron regions (Supplementary Fig. 5b). Metagene analysis revealed that the m^6^A sites were predominantly localized near the translation stop codons (Supplementary Fig. 5c). Furthermore, we found that *Pth1r*, a critical regulator of lineage allocation in MSCs and osteoblast precursors^34–36^, has high enriched and specific m^6^A peak near its translation stop codon (Fig. 5a, b).

Next, we carried out RNA sequencing (RNA-seq) to examine transcriptome profiles in MSCs isolated from *Prx1-Cre;Mettl3*^*fl/fl*^ mice and *Mettl3*^*fl/fl*^ controls, and confirmed the down-regulated expression of osteogenic marker genes, such as *Dlx5*, *Sp7*, *Alpl*, and *Bglap*, whereas upregulated expression of markers favoring adipocytic differentiation, including *Pparg*, *Plin1*, and *Fabp4* (Fig. 5c). PTH1R is a key receptor of PTH and a parathyroid hormone-related protein (PTHrP) analog, abaloparatide, both are FDA-approved anabolic drugs for the treatment of osteoporosis^37,38^, and is indispensable for PTH’s function in modulating bone homeostasis^39,40^. To confirm that m^6^A modulates Pth1r, we preceded our RNA-seq data to gene set enrichment analysis (GSEA) using a published list of PTH-regulated genes^41^. We found that *Mettl3*-deficient MSCs underwent a global down-regulation of PTH-regulated gene expression (Fig. 5d), implying PTH/Pth1r signaling axis as a potential mechanism for Mettl3-mediated m^6^A methylation on MSCs fate.

Both RNA-seq and quantitative RT-PCR showed that the mRNA expression of *Pth1r* was hardly affected by *Mettl3* deletion (Fig. 5e), whereas the Pth1r protein synthesis was inhibited upon *Mettl3* deficiency as evidenced by western blot and decreased translation efficiency (Fig. 5f, g), suggesting that m^6^A methylation affected Pth1r expression at translation level. We subsequently performed sucrose gradient centrifugation to resolve polysome fractions of *Mettl3* knockout and control MSCs, and used RT-PCR to examine the distribution of endogenous *Pth1r* mRNA in different ribosome fractions. Our data revealed that the relative distribution of *Pth1r* mRNA was shifted from the polysome fractions to the sub-polysome fractions in *Mettl3* knockout cells, indicating that *Mettl3* depletion results in the decreased translation efficiency of *Pth1r* mRNA (Fig. 5h, i).

### m^6^A is essential for PTH function

**Fig. 6:**
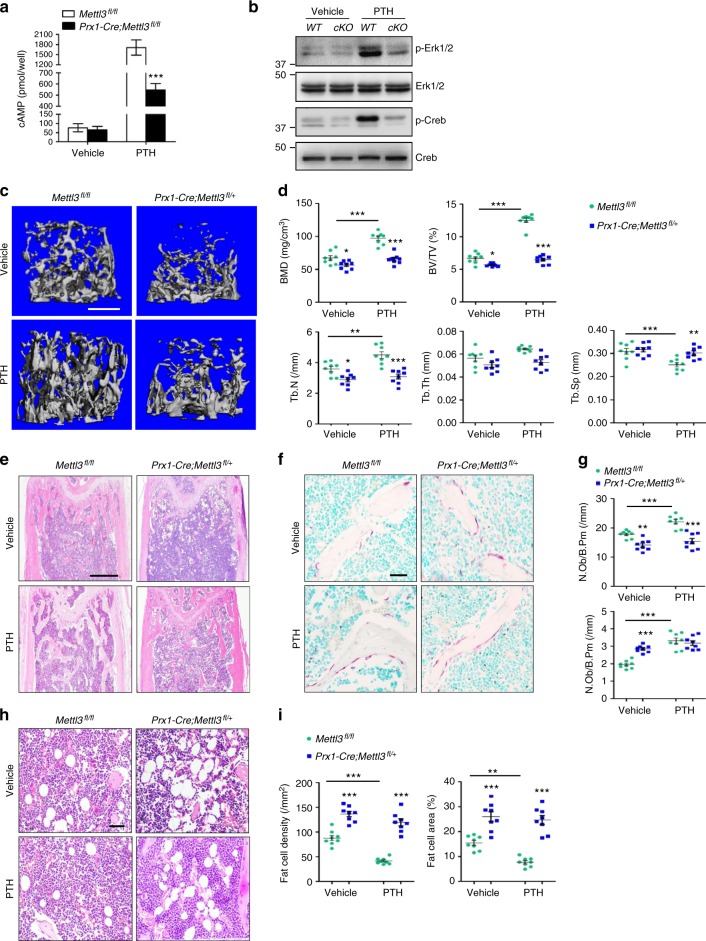
Deletion of *Mettl3* exhibits inert responses to PTH treatment. **a** PTH-induced accumulation of cAMP in MSCs. Results are from three independent experiments. **b** Western blot analysis of p-ERK1/2, ERK1/2, p-CREB, and CREB. *WT*: *Mettl3*^*fl/fl*^, *cKO*: *Prx1-Cre;Mettl3*^*fl/fl*^. **c** Representative μCT images of distal femurs following PTH treatment. **d** Quantitative μCT analyses of metaphysis region of distal femurs (*n* = 8). **e** H&E staining of femoral sections from *Mettl3*^*fl/fl*^ and *Prx1-Cre;Mettl3*^*fl/+*^ female mice following PTH treatment. Scale bar, 500 μm. **f** TRAP staining of femur sections. Scale bar, 50 μm. **g** Histomorphometric analyses of distal femurs following PTH treatment (*n* = 8). **h** Representative H&E staining of femur sections exhibiting the marrow adipose tissue. Scale bar, 50 μm. **i** Number and area of adipocytes in the distal marrow per tissue area (*n* = 8). Data are expressed as mean ± s.e.m.; *P < 0.05, **P < 0.01, ***P < 0.001 by two-way ANOVA with Bonferroni post tests

Since Pth1r protein synthesis was hampered by *Mettl3* depletion, we next examined physiological functions of PTH/Pth1r axis upon *Mettl3* deficiency. Cyclic AMP-dependent protein kinase A (PKA) and extracellular signal-regulated kinases (ERK) pathways are important downstream pathways that mediate the intracellular signals and physiological responses during the anabolic effect of PTH^42,43^. Loss of *Mettl3* attenuated the PTH-induced cAMP accumulation of *Prx1-Cre;Mettl3*^*fl/fl*^ MSCs (Fig. 6a) and consequently inhibited the downstream phosphorylation of cAMP response element-binding protein (CREB) (Fig. 6b). Meanwhile, despite having the same basal level of ERK1/2 as the control MSCs, mutant MSCs failed to activate the ERK1/2 pathway after PTH treatment (Fig. 6b).

To investigate the potential role of Mettl3 in PTH-mediated bone remodeling in vivo, we performed intermittent PTH (1-34) injection to 3-month-old *Mettl3*^*fl/fl*^ and *Prx1-Cre;Mettl3*^*fl/+*^ female mice to compare their responses to PTH treatment. In contrast with the sharp growth of trabecular bone mass in *Mettl3*^*fl/fl*^ controls, there were only slight changes in the BMD, BV/TV, Tb.N and Tb.Sp in the *Prx1-Cre;Mettl3*^*fl/+*^ mice (Fig. 6c, d). Furthermore, *Prx1-Cre;Mettl3*^*fl/+*^ mice failed to undergo an effective increase in bone remodeling after PTH treatment (Fig. 6e–g), confirming that *Mettl3* knockout blunted PTH-induced osteogenic effects. Besides a marked enrichment in osteogenesis, PTH also inhibits adipocytic differentiation to promote bone formation^35,44^. In our experiment, PTH injection significantly reduced MAT accumulation in *Mettl3*^*fl/fl*^ mice whilst hardly reversing the high marrow adiposity in *Prx1-Cre;Mettl3*^*fl/+*^ mice (Fig. 6h, i), consolidating our findings that m^6^A affects both osteogenic and adipogenic differentiation of MSCs by regulating PTH/Pth1r signaling axis.

### Overexpression of ***Pth1r*** partially rescues the proper lineage commitment of ***Mettl3***-deficient MSCs

**Fig. 7:**
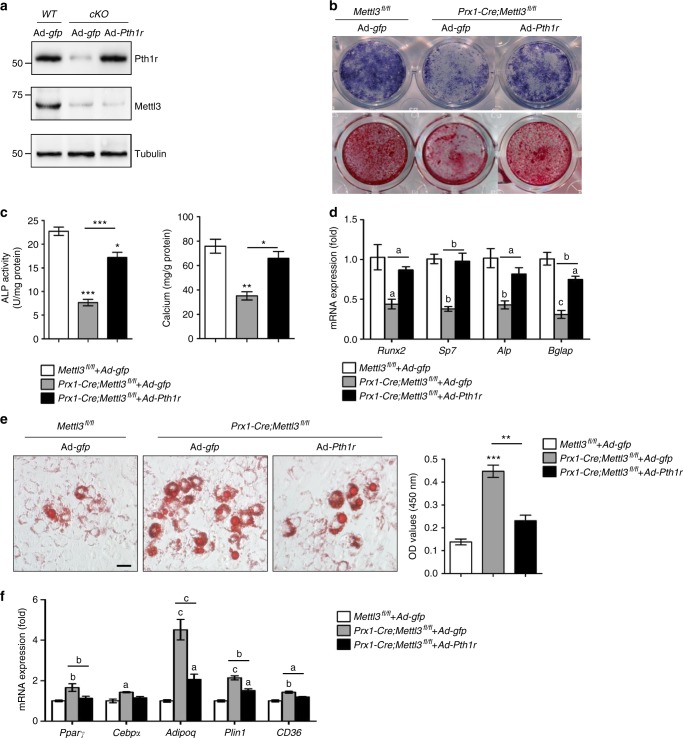
Overexpression of *Pth1r* ameliorates osteogenic differentiation of *Mettl3*-deficient MSCs. **a** Western blot analysis of Pth1r and Mettl3. *WT*: *Mettl3*^*fl/fl*^, *cKO*: *Prx1-Cre;Mettl3*^*fl/fl*^. **b**, **c** Representative images and quantitative analyses of ALP and ARS staining. **d** qRT-PCR analyses of the expression of *Runx2*, *Sp7*, *Alp*, and *Bglap* under osteogenic condition. **e** Representative images and quantitative analysis of oil red O staining. Scale bar, 100 μm. **f** qRT-PCR analyses of the expression of *Cebpα*, *Pparγ*, *Adipoq, Plin1* and *CD36* under adipogenic condition. Results are from three independent experiments. Data are expressed as mean ± s.e.m.; *P < 0.05, **P < 0.01, ***P < 0.001 (**c**, **e**) and a: *P* < 0.05, b: *P* < 0.01, c: *P* < 0.001 (**d**, **f**) by one-way ANOVA with Tukey’s post hoc test

Next, we transduced adenoviral constructs overexpressing *Pth1r* (Ad-*Pth1r*), or *gfp* (Ad-*gfp*) as a control, into *Prx1-Cre;Mettl3*^*fl/fl*^ MSCs. Ad-*Pth1r* successfully restored the protein level of Pth1r in *Prx1-Cre;Mettl3*^*fl/fl*^ MSCs (Fig. 7a), and significantly improved the ALP activity and calcium nodule formation (Fig. 7b, c). Furthermore, *Pth1r* overexpression ameliorated the increased intensity of oil red O staining arising from m^6^A reduction (Fig. 7e). qRT-PCR analysis also revealed an up-regulation of osteogenic markers along with a down-regulation of adipogenic markers after *Pth1r* transduction (Fig. 7d, f). These data demonstrate that *Pth1r* overexpression is able to partially rescue the proper lineage commitment of *Mettl3*-deficient MSCs.

## Discussion

Mettl3-mediated m^6^A modification in eukaryotic RNA has a broad functional influence on stabilizing homeostasis, while any perturbation in m^6^A levels can result in malfunction or diseases. Recent studies have consecutively revealed a strong and intricate relation of m^6^A modification with stem cell regulation. Mis-regulated m^6^A level disrupted the timely controlled embryonic stem cell differentiation^20,21^, impaired the normal generation and differentiation of hematopoietic stem cells^23,24^ as well as inhibiting functional development of neurogenesis, spermatogenesis, and T cell homeostasis^25–28,45^. Using different temporal MSCs markers, *Prx1* and *Lepr*, we generated two genetic murine models to conditionally knockout *Mettl3* from early limb bud mesenchyme and adult bone marrow, respectively. We showed that deletion of *Mettl3* in bone marrow MSCs disrupts cell fate in mice and results in osteoporosis pathological phenotypes such as decreased bone mass with incompetent osteogenic potential, and increased marrow adiposity with enhanced adipogenic potential, uncovering an efficient and specific regulation of m^6^A on MSCs. Furthermore, *Mettl3* gain-of-function prevents estrogen deficiency-induced postmenopausal osteoporosis, establishing the indispensability of Mettl3 in defining bone marrow MSC’s fate and thereby ensuring skeletal health.

Accumulating evidences have pointed out the regulatory role of m^6^A in translation. In cooperation with m^6^A readers, m^6^A facilitates translation initiation, and responds to selective mRNA translation under heat shock stress^14,16,46–48^. Alterations in translation efficiency of m^6^A marked transcripts affected normal hematopoiesis^23,24^ and mammalian spermatogenesis^28,49^. Here we discover that m^6^A is essential for Pth1r translation. Loss of *Mettl3* caused strong shift of *Pth1r* mRNA from the polysome fractions to the sub-polysome fractions, therefore slowing down the protein synthesis of Pth1r and further blocking the downstream signaling pathways of Pth1r responsive to PTH treatment.

Intermittent PTH treatment promotes bone formation, which is achieved by increasing osteoblasts number, delaying osteoblast apoptosis, activating bone-lining cells and reducing marrow fat^34,42,44^. PTH1R is a direct signaling mediator of PTH in promoting osteoblastic lineage of MSCs while aberrant PTH1R expression can lead to MAT accumulation as a consequence of preferential adipogenic differentiation^34,35,50^. *Mettl3* knockout reduced the global methylation level of m^6^A in mice and significantly impaired Pth1r function. The persistence in high marrow adiposity and the poorly elevated bone mass in m^6^A deficient mice following intermittent PTH(1-34) injection supported a blunt response to PTH anabolic effect. Our subsequent finding that overexpression of *Pth1r* could largely reverse skewed MSCs differentiation towards adipocytes and improve osteogenesis further confirmed PTH/Pth1r signaling pathway as a novel mechanism whereby Mettl3-mediated m^6^A modification controls the lineage allocation of MSCs.

PTH also stimulates osteoclast formation and activity by increasing the receptor activator of nuclear factor κ-B ligand (RANKL)/osteoprotegerin (OPG) ratio through its receptor^51–53^. Although the protein synthesis of Pth1r was largely compromised upon *Mettl3* ablation in our study, we found that the number of osteoclasts was rather elevated. One explanation for this increase in osteoclasts, we presume, is that Mettl3 has another relevant target in bone cells besides Pth1r, since our overexpression of *Pth1r* has only achieved a partial rescue. Another possible explanation is that the adipogenic favored differentiation of MSCs upon Pth1r malfunction promotes osteoclast activity. Fan et al. demonstrated in their recent study that bone marrow adipocytes are the source of RANKL in the absence of PTH1R signaling^34^. Similar to their finding, our TRAP staining showed that osteoclasts in *Mettl3* mutant mice were located very near to the large adipocytes (Fig. 1d). Therefore, we speculated that the excessive accumulated marrow adipose tissue in *Mettl3* conditional knockout mice may also account for the increase of osteoclast number.

Of note, unlike the severe chondrodysplasia in *Prx1-Cre;PTH1R*^*fl/fl*^ mice reported by Fan et al.^54^, we observed only slight yet not significant growth plate cellular architecture changes in *Prx1-Cre;Mettl3*^*fl/fl*^ mice. Our finding that Mettl3 does not express in growth plate chondrocytes, thus may not participate in chondrogenesis, can be an explanation for this discrepancy in growth plate phenotype. The minute difference in hypertrophic zone between *Prx1-Cre;Mettl3*^*fl/fl*^ mice and their controls might due to an indirect effect of Pth1r impairment in *Mettl3*-depleted osteoblasts, which further affects endochondral vascularization in the growth plate^55^.

In summary, we demonstrate that reduced epigenetic modification of m^6^A in bone marrow MSCs from an early phase inhibits Pth1r translation in mammals and blocks their anabolic responses to PTH during bone accrual. These functional defects tilt the delicate MSC differentiation balance toward adipogenic lineage, causing severe bone loss and excessive MAT accumulation. Our data enrich the evidence of m^6^A in modulating stem cell differentiation, and uncover the functional link between mis-regulated m^6^A modification and bone pathological disorders, therefore may shed light on the development of new therapeutic strategies for osteoporosis.

## Methods

### **Generation of conditional*Mettl3*knockout and knock-in mice**

For conditional knockout, *Mettl3*^*f*l/+^ mice with C57BL6/J background were generated using CRISPR-Cas9 systems^56,57^. Briefly, the exon 2 and exon 3 of *Mettl3* is the target of interest in generation of conditional knockout floxed *Mettl3* allele. With the help of a CRISPR design tool (http://crispr.mit.edu), sgRNAs were designed to recognize a region either upstream or downstream of the target, and then were screened for on-target activity using a Universal CRISPR Activity Assay (UCA^TM^, Biocytogen Inc, Beijing). Donor vectors structured with the Lox P flanked exon 2 and exon 3 was mixed with Cas9 mRNA and sgRNAs and co-injected into the cytoplasm of one-cell stage fertilized C57BL6/J eggs. The injected zygotes were then transferred into oviducts of Kunming pesudopregnant females to obtain F0 mice. Tail genomic DNA PCR and sequencing were performed to identify F0 mice with expected genotype, which were afterwards crossed with C57BL6/J mice to establish germline-transmitted F1 founders. Both PCR genotyping and southern blot were applied to ensure the correct genotype of generated *Mettl3*^*fl/+*^ mice.

For conditional knock-in, CRISPR-Cas9 techniques were also performed. The targeting vector consisting of a loxP flanked stop codon regulatory element and a downstream *Mettl3* coding sequence (CDS) was inserted into wild type *Rosa26* allele to obtain the targeted conditional *Mettl3* knock-in allele. We designed a *tdTomato* sequence at the downstream of *Mettl3* CDS to facilitate our verification of knock-in efficiency later in the research. The targeted allele was then reconstructed into a plasmid under the mediation of Cas9/sgRNAs. After the co-injection of the reconstructed plasmid into fertilized C57BL6/J eggs, PCR genotyping and southern blot were executed to obtain desirable F1 mice that were recombined with targeted *Mettl3* knock-in alleles.

*Prx1-Cre* and *Lepr-Cre* transgenic mice were purchased from The Jackson Laboratory. We crossed male *Prx1-Cre* or *Lepr-Cre* mice with *Mettl3*^*f*l/+^ mice to obtain *Prx1-Cre;Mettl3*^*fl/+*^ mice and *Lepr-Cre;Mettl3*^*fl/+*^ mice as heterozygous conditional *Mettl3* knockout. By mating *Prx1-Cre;Mettl3*^*fl/+*^ or *Lepr-Cre;Mettl3*^*fl/+*^ male mice with *Mettl3*^*f*l/+^ female mice, we obtained *Prx1-Cre;Mettl3*^*fl/fl*^ mice and *Lepr-Cre;Mettl3*^*fl/fl*^ mice as homozygous conditional *Mettl3* knockout mice. The same mating strategy was used in the generation of conditional *Mettl3* knock-in mice.

The genotype of transgenic mice was identified by PCR analyses of genomic DNA extracted from mouse tails. Primers for floxed *Mettl3* knockout allele genotyping were forward (5′-TCCCTGGGAAACATAATCACATCC-3′) and reverse (5′-CTTCTCCTCCTCTCAAATGATACAAT-3′). Primers for floxed *Mettl3* knock-in allele genotyping were forward (5′-AGTCGCTCTGAGTTGTTATCAG-3′), wild-type band reverse (5′-TGAGCATGTCTTTAATCTACCTCGATG-3′), mutant band reverse (5′- AGTCCCTATTGGCGTTACTATGG-3′). Primers for *Cre* transgene genotyping were wild-type band forward (5′-CCATCTGCCACCAGCCAG-3′), wild-type band reverse (5′-TCGCCATCTTCCAGCAGG-3′), mutant band forward (5′-ACTGGGATCTTCGAACTCTTTGGAC-3′), and mutant band reverse (5′-GATGTTGGGGCACTGCTCATTCACC-3′). All the mice were bred and maintained under specific-pathogen-free conditions. All studies performed were approved by the Subcommittee on Research and Animal Care (SRAC) of Sichuan University.

### LC-MS/MS analysis of m^6^A level

The integrity and quantity of each total RNA samples extracted from primary MSCs was examined using agarose gel electrophoresis and Nanodrop™ instrument. mRNA was isolated from total RNA using NEBNext® Poly(A) mRNA Magnetic Isolation Module (For next generation sequencing using). Purified mRNA was quantified using NanoDrop ND-1000. mRNA was hydrolyzed to single nucleosides and then nucleosides were dephosphorylated by enzyme mix. Pretreated nucleosides solution was deproteinized using Satorius 10,000-Da MWCO spin filter.

Analysis of nucleoside mixtures was performed on Agilent 6460 QQQ mass spectrometer with an Agilent 1290 HPLC system. Multi reaction monitoring (MRM) mode was performed because of its high selectivity and sensitivity attained working with parent-to-product ion transitions. LC-MS/MS data was acquired using Agilent Qualitative Analysis software. MRM peaks of each modified nucleoside were extracted and normalized to peak areas of normal adenosine in each samples. Samples were run in duplicate, and m^6^A/A ratios were calculated.

### μCT and histomorphometric analyses

The harvested bone tissues were fixed in 4% polyoxymethylene for 2 days and then stored in 70% ethanol at 4 °C before being processed. μCT analysis was performed according to recent guidelines^58^ using a μCT 80 microCT system (Scanco Medical, Bassersdorf, Switzerland) with a spatial resolution of 8 μm (55 kV, 114 mA, 500 ms integration time). For the analysis of cortical bone regeneration, the volume of interest (VOI) was defined as a cylindrical area covering the initial bone defect. Bone volume (BV/TV) was calculated within the delimited VOI.

Processing of undecalcified bone specimens and cancellous bone histomorphometry were performed as described^59,60^. Followed by microCT scanning, femurs were dehydrated and embedded in methylmethacrylate. Five‐µm‐thick sections were prepared using a Leica RM2235 microtome and were stained by the von Kossa/nuclear fast red method. Histomorphometric measurements in the distal end of femurs were made using OsteoMeasure software (OsteoMetrics, Decatur, GA).

### Immunohistochemical staining

The dissected femurs were fixed in 4% polyoxymethylene for 2 days and decalcified in 10% EDTA for 2 weeks before sectioning (5 μm). Slides were subjected to sodium citrate buffer at 99 °C for 20 min for antigen retrieval and then incubated with rabbit anti-METTL3/MT-A70 (1:200, Bethyl, Cat No: A301-567A), or rabbit polyclonal anti-FABP4 (1:200, Abcam, Cat No: ab13979).

### Ovariectomy

Nine-week-old female conditional *Mettl3* knock-in mice and littermate controls were selected for ovariectomy and randomly assigned to OVX or sham group. Ovaries were surgically removed on both sides after anesthesia^61^. In short, small dorsal incisions below the bottom of the rib cage were made to penetrate the skin and muscles respectively. After locating the ovary, we exposed the oviduct and ligated it before the removal of the ovary. For sham group, ovaries were replaced back into the peritoneal cavity directly after their locating. Finally, we sutured the wound and bred the operated mice in sham and OVX group for another 5 weeks before sacrifice.

### Cell culture

We isolated primary MSCs by flushing the bone marrow of tibiae and femurs. The cells were then cultured in α-MEM (Hyclone) supplemented with 10% fetal bovine serum (Gibco),100 units/ml penicillin (Gibco) and 100 μg/ml streptomycin (Gibco), at 37 °C with an atmosphere of 5% CO_2_ and 95% humidity. For osteogenic induction, MSCs were seeded in six-well plates and treated with osteogenic medium containing 50 μg/ml ascorbic acid, 5 mM β-glycerophosphate, and 100 nM dexamethasone (all from Sigma). For adipogenic induction, MSCs were seeded in six-well plates and treated with adipogenic medium containing 0.5 μM isobutylmethylxanthin (Sigma), 10 μg/ml insulin (Sigma), and 1 μM dexamethasone (Sigma).

### ALP, alizarin red staining, and oil red O staining

For ALP staining, cells were fixed after 10 days induction of osteogenic differentiation with 4% polyoxymethylene for 15 min and incubated with 0.1 M Tris buffer (pH 9.3) containing 0.25% naphthol AS-BI phosphate (Sigma) and 0.75% Fast Blue BB (Sigma). Quantitative ALP activity was performed with a commercial kit according to the manufacturer’s protocol (Cell Biolab, San Diego, CA). The optical density was determined by a spectrophotometer (Thermo Fisher Scientific) at 450 nm.

For alizarin red staining (ARS) staining, cells were fixed after 10 days induction of osteogenic differentiation with 4% polyoxymethylene for 15 min and stained with 1% Alizarin red S (pH 4.2, Sigma-Aldrich) for 5 min. Mineralized matrix stained with alizarin red were destained with 10% cetylpyridinium chloride in 10 mM sodium phosphate (pH 7.0), and the calcium concentration was determined by a spectrophotometer (Thermo Fisher Scientific) at 562 nm using a standard calcium curve in the same solution^62,63^.

For oil red O staining, after 2–3 weeks of adipogenic induction, cells were subjected to oil red O staining (Sigma) according to the manufacturer’s protocols. The staining was then dissolved with isopropanol and quantified by a spectrophotometer (Thermo Fisher Scientific) at 450 nm, as described previously^34^.

### Quantitative RT-PCR

Total RNA of cultured cells was extracted using Trizol (Invitrogen) according to manufacturer’s instruction. cDNA was prepared using PrimeScript RT reagent Kit with gDNA Eraser (Takara). The RNA concentration was measured with a NanoDrop 2000 (Thermo Fisher Scientific). qRT-PCR was performed using SYBR Premix Ex Taq II (Takara) in CFX96 Real-Time System (Bio-Rad). Relative gene expression was normalized by *Gapdh* for osteogenic differentiation and *36B4* for adipogenic differentiation using a 2^−ΔΔCt^ method. The primers are listed in Supplementary Table 1.

### Western blot

Cells were lysed in RIPA buffer (Pierce, Rockford, IL) on ice. For PTH treatment group, cells were given 100 nM human recombinant PTH (1-34) (Bachem, Torrance, CA, USA) at 37 °C for 30 min prior to sampling. The samples were heated at 95 ℃ for 5 min in sample buffer containing 2% SDS and 1% 2-mercaptoethanol, separated on 10% SDS–polyacrylamide gels, and transferred to PVDF membranes by a wet transfer apparatus (Bio-Rad). The membranes were blotted with 5% BSA for 1 h and then incubated overnight with rabbit METTL3 polyclonal antibody (1:1000, Proteintech, Cat No: 15073-1-AP), PTH1R antibody (1:1000, Assay Biotechnology, Cat No: G220), p44/42 MAPK (Erk1/2) rabbit mAb (1:1000, Cell signaling, Cat No: 4695), phospho-p44/42 MAPK (Erk1/2) rabbit mAb (1:2000, Cell signaling, Cat No: 4370), CREB rabbit mAb (1:1000, Cell signaling, Cat No: 9197), phospho-CREB rabbit mAb (1:1000, Cell signaling, 9198), α-tubulin rabbit polyclonal antibody (1:2000, Beyotime, Cat No: AF0001), followed by incubation with a goat anti-rabbit IgG secondary antibody HRP conjugated (1:5000, Signaling antibody, Cat No: L3012-2). The antibody-antigen complexes were visualized with Immobilon reagents (Millipore). The uncropped gel images are shown in Supplementary Fig. 6.

### m^6^A MeRIP-Seq (methylated RNA immunoprecipatation and sequencing)

The MSC m^6^A MeRIP-Seq and data analysis were performed as previously described^64,65^. Briefly, Trizol reagent was used for the isolation of total RNA from MSC, and then the PolyATtract mRNA Isolation System IV (Promega Z5310) was used to enrich mRNA from MSC total RNA sample. Two μg of the purified mRNA was fragmentized into about 100 nt length with ZnCl_2_ buffer at 94 °C for 5 min. The mRNA fragments were purified with Oligo Clean & Concentrator^TM^ (Zymoresearch D4061) and then subjected to immunoprecipitation with α-m^6^A antibody (1:100, Synaptic Systems, Cat No: 202003). After extensive wash, the methylated fragments were eluted by competition using free N^6^-Methyladenosine (Santa Cruz Biotechnology, sc-215524) and then used for library construction with the TruSeq Stranded mRNA Sample Prep Kits (Illumina RS-122-2101). The input and m^6^A MeRIP libraries were sequenced with Illumina NextSeq 500 with high output kit. The sequence reads mapping, peak calling and visualization, metagene analysis of m^6^A distribution, motif search and gene ontology analysis were performed as previously described^65^.

### RNA-seq and gene set enrichment analysis

Total RNAs from MSCs of *Prx1-Cre;Mettl3*^*fl/fl*^ mice and their control littermates were extracted by Trizol reagent and purified using poly-T oligo-attached magnetic beads. Sequencing libraries were generated using NEBNext® Ultra™ RNA Library Prep Kit for Illumina® (NEB, USA) following manufacturer’s recommendations, and were then subjected to Illumina HiSeq 2500. We used FastQC (v0.11.5) and FASTX toolkit (0.0.13) to control the quality of RNA-seq data and mapped them to Mus musculus reference genomes using HISAT2 (v.2.0.4). Ballgown software (v.3.4.0) was performed to identify differentially expressed genes and transcripts. Genes were considered significantly differentially expressed if showing ≥2.0 fold change and *P* value < 0.05.

For GSEA, PTH-regulated gene list was obtained from a published study^41^. GSEA were performed with GSEA software (http://www.broad.mit.edu/GSEA) as previously described^66^. In short, we first imported our gene list of interest into the software and examined whether this given gene set showed statistically significant, concordant differences between two biological states (for example, phenotypes). *P* values were computed using a bootstrap distribution created by resampling gene sets of the same cardinality.

### Polysome fractionation and RT-PCR

Cells were washed with ice old PBS twice and then PBS containing 100 μg/ml Cycloheximide was added to the cell culture dishes to stop translation. Cells were scraped and lyzed with cell extraction buffer to collect the cytoplasmic extracts, which were loaded onto the top of 11 ml 10–50% sucrose gradient tube. The sucrose gradient tubes were centrifuged at 222,000 × *g* with SW41Ti rotor for 2 h and 30 min at 4 °C. Then the ribosome fractions were collected using the ISCO gradient fractionators, with measurement of absorbance at 254 nm. RNA samples were isolated from different fractions using Trizol and reverse transcription and PCR were performed as previously described to determine the relative distribution of *Pth1r* mRNA in different ribosome fractions^65^.

### cAMP assay

Cells were cultured in six-well plates and stimulated with vehicle or 100 nM human recombinant PTH (1-34) (Bachem, Torrance, CA, USA) at 37 °C for 15 min^67^. We then carried out the cAMP assay using the cAMP Biotrak Enzymeimmunoassay (EIA) System (GE Healthcare Bio-Science Corp., Piscataway, NJ, USA) and followed the manufacturer’ s instruction.

### Intermittent PTH (1-34) injection

Subcutaneous daily injection of 80 μg/kg of human recombinant PTH (1-34) (Bachem, Torrance, CA, USA) was given to 12-week-old female *Prx1-Cre;Mettl3*^*fl/+*^ and control mice for 4 weeks. An equal volume of sterile saline was given to animals in the vehicle group. The assignment of *Prx1-Cre;Mettl3*^*fl/+*^ and control mice into PTH or vehicle group was randomized.

### **Adenoviral infection**

Adenoviruses encoding mouse *Pth1r* gene and/or green fluorescent protein (GFP) were obtained from Cyagen. Primary MSCs isolated from bone marrow of tibiae and femurs were infected with Ad-*Pth1r* or Ad-*gfp* for 12 h at 37 °C with an atmosphere of 5% CO_2_ and 95% humidity.

### Statistical analysis

All data were expressed as mean ± s.e.m. Statistical differences were analyzed by unpaired two-tailed Student’s *t* test for comparison between two groups, one-way ANOVA followed by Tukey’s post hoc test for multiple comparisons, and two-way ANOVA followed by Bonferroni post test for comparison between sham and OVX groups, as well as vehicle and PTH treatment groups. A *P* value <0.05 was considered to be statistically significant.

## Electronic supplementary material

Supplementary Information

Supplementary Data 1

Description of Additional Supplementary Files

## Data Availability

All Seq data have been deposited into NCBI database with the identifier GSE114933.

## References

[1] Rosen CJ, Bouxsein ML (2006). Mechanisms of disease: is osteoporosis the obesity of bone?. Nat. Clin. Pract. Rheum..

[2] Devlin MJ, Rosen CJ (2015). The bone–fat interface: basic and clinical implications of marrow adiposity. Lancet Diabetes Endocrinol..

[3] Kawai M, Devlin MJ, Rosen CJ (2009). Fat targets for skeletal health. Nat. Rev. Rheumatol..

[4] Rosen CJ, Ackertbicknell C, Rodriguez JP, Pino AM (2009). Marrow fat and the bone microenvironment: developmental, functional, and pathological implications. Crit. Rev. Eukar. Gene.

[5] Scheller EL, Rosen CJ (2014). What’s the matter with MAT? Marrow adipose tissue, metabolism, and skeletal health. Ann. NY Acad. Sci..

[6] Liu J (2014). A METTL3-METTL14 complex mediates mammalian nuclear RNA N6-adenosine methylation. Nat. Chem. Biol..

[7] Yue Y, Liu J, He C (2015). RNA N6-methyladenosine methylation in post-transcriptional gene expression regulation. Gene. Dev..

[8] Wang X (2014). N6-methyladenosine-dependent regulation of messenger RNA stability. Nature.

[9] Wang P, Doxtader KA, Nam Y (2016). Structural basis for cooperative function of Mettl3 and Mettl14 methyltransferases. Mol. Cell.

[10] Wang X (2016). Structural basis of N(6)-adenosine methylation by the METTL3-METTL14 complex. Nature.

[11] Ping XL (2014). Mammalian WTAP is a regulatory subunit of the RNA N6-methyladenosine methyltransferase. Cell Res..

[12] Jia G (2011). N6-methyladenosine in nuclear RNA is a major substrate of the obesity-associated FTO. Nat. Chem. Biol..

[13] Zheng G (2013). ALKBH5 is a mammalian RNA demethylase that impacts RNA metabolism and mouse fertility. Mol. Cell.

[14] Zhou J (2015). Dynamic m(6)A mRNA methylation directs translational control of heat shock response. Nature.

[15] Dominissini D (2012). Topology of the human and mouse m6A RNA methylomes revealed by m6A-seq. Nature.

[16] Wang X (2015). N6-methyladenosine modulates messenger RNA translation efficiency. Cell.

[17] Lence T (2016). m(6)A modulates neuronal functions and sex determination in Drosophila. Nature.

[18] Zhang S (2017). m6A demethylase ALKBH5 maintains tumorigenicity of glioblastoma stem-like cells by sustaining FOXM1 expression and cell proliferation program. Cancer Cell..

[19] Barbieri I (2017). Promoter-bound METTL3 maintains myeloid leukaemia by m(6)A-dependent translation control. Nature.

[20] Geula S (2015). m6A mRNA methylation facilitates resolution of naive pluripotency toward differentiation. Science.

[21] Batista PJ (2014). m(6)A RNA modification controls cell fate transition in mammalian embryonic stem cells. Cell Stem Cell.

[22] Zhang C (2017). m6A modulates haematopoietic stem and progenitor cell specification. Nature.

[23] Weng H (2018). METTL14 inhibits hematopoietic stem/progenitor differentiation and promotes leukemogenesis via mRNA m(6)A modification. Cell Stem Cell.

[24] Vu LP (2017). The N6-methyladenosine (m6A)-forming enzyme METTL3 controls myeloid differentiation of normal hematopoietic and leukemia cells. Nat. Med..

[25] Yoon KJ (2017). Temporal control of mammalian cortical neurogenesis by m6A methylation. Cell.

[26] Li HB (2017). m6A mRNA methylation controls T cell homeostasis by targeting the IL-7/STAT5/SOCS pathways. Nature.

[27] Xu K (2017). Mettl3-mediated m6A regulates spermatogonial differentiation and meiosis initiation. Cell Res..

[28] Lin Z (2017). Mettl3-/Mettl14-mediated mRNA N6-methyladenosine modulates murine spermatogenesis. Cell Res..

[29] Haussmann IU (2016). m(6)A potentiates Sxl alternative pre-mRNA splicing for robust Drosophila sex determination. Nature.

[30] Zhou BO, Yue R, Murphy MM, Peyer JG, Morrison SJ (2014). Leptin-receptor-expressing mesenchymal stromal cells represent the main source of bone formed by adult bone marrow. Cell Stem Cell.

[31] Yue R, Zhou BO, Shimada IS, Zhao Z, Morrison SJ (2016). Leptin receptor promotes adipogenesis and reduces osteogenesis by regulating mesenchymal stromal cells in adult bone marrow. Cell Stem Cell.

[32] Kalu DN (1991). The ovariectomized rat model of postmenopausal bone loss. Bone Miner..

[33] Lewiecki EM (2011). New targets for intervention in the treatment of postmenopausal osteoporosis. Nat. Rev. Rheumatol..

[34] Fan Y (2017). Parathyroid hormone directs bone marrow mesenchymal cell fate. Cell. Metab..

[35] Balani DH, Ono N, Kronenberg HM (2017). Parathyroid hormone regulates fates of murine osteoblast precursors in vivo. J. Clin. Invest..

[36] Wanida O, Naoko S, Shigeki N, Noriaki O, Kronenberg HM (2016). Parathyroid hormone receptor signalling in osterix-expressing mesenchymal progenitors is essential for tooth root formation. Nat. Commun..

[37] Neer RM (2001). Effect of parathyroid hormone (1-34) on fractures and bone mineral density in postmenopausal women with osteoporosis. N. Engl. J. Med..

[38] Miller PD (2016). Effect of abaloparatide vs placebo on new vertebral fractures in postmenopausal women with osteoporosis: a randomized clinical trial. JAMA.

[39] Jüppner H (1991). A G protein-linked receptor for parathyroid hormone and parathyroid hormone-related peptide. Science.

[40] Abousamra AB (1992). Expression cloning of a common receptor for parathyroid hormone and parathyroid hormone-related peptide from rat osteoblast-like cells: a single receptor stimulates intracellular accumulation of both cAMP and inositol trisphosphates and increases intracell. Proc. Natl Acad. Sci. USA.

[41] Qin L (2003). Gene expression profiles and transcription factors involved in parathyroid hormone signaling in osteoblasts revealed by microarray and bioinformatics. J. Biol. Chem..

[42] Jilka RL (2007). Molecular and cellular mechanisms of the anabolic effect of intermittent PTH. Bone.

[43] Gesty-Palmer D (2006). Distinct beta-arrestin- and G protein-dependent pathways for parathyroid hormone receptor-stimulated ERK1/2 activation. J. Biol. Chem..

[44] Rickard DJ (2006). Intermittent treatment with parathyroid hormone (PTH) as well as a non-peptide small molecule agonist of the PTH1 receptor inhibits adipocyte differentiation in human bone marrow stromal cells. Bone.

[45] Wang Y (2018). N6-methyladenosine RNA modification regulates embryonic neural stem cell self-renewal through histone modifications. Nat. Neurosci..

[46] Shi H (2017). YTHDF3 facilitates translation and decay of N6-methyladenosine-modified RNA. Cell Res..

[47] Li A (2017). Cytoplasmic m6A reader YTHDF3 promotes mRNA translation. Cell Res..

[48] Yang Y (2017). Extensive translation of circular RNAs driven by N(6)-methyladenosine. Cell Res..

[49] Hsu PJ (2017). Ythdc2 is an N6-methyladenosine binding protein that regulates mammalian spermatogenesis. Cell Res..

[50] Calvi LM (2001). Activated parathyroid hormone/parathyroid hormone-related protein receptor in osteoblastic cells differentially affects cortical and trabecular bone. J. Clin. Invest..

[51] Silva BC, Costa AG, Cusano NE, Kousteni S, Bilezikian JP (2011). Catabolic and anabolic actions of parathyroid hormone on the skeleton. J. Endocrinol. Invest..

[52] Silva BC, Bilezikian JP (2015). Parathyroid hormone: anabolic and catabolic actions on the skeleton. Curr. Opin. Pharmacol..

[53] Wein Marc N., Kronenberg Henry M. (2018). Regulation of Bone Remodeling by Parathyroid Hormone. Cold Spring Harbor Perspectives in Medicine.

[54] Fan Y (2016). Parathyroid hormone 1 receptor is essential to induce FGF23 production and maintain systemic mineral ion homeostasis. FASEB J..

[55] Qiu T (2015). PTH receptor signaling in osteoblasts regulates endochondral vascularization in maintenance of postnatal growth plate. J. Bone Miner. Res..

[56] Mali P (2013). RNA-guided human genome engineering via Cas9. Science.

[57] Zhang F (2013). Multiplex genome engineering using CRISPR/Cas systems. Science.

[58] Bouxsein ML (2010). Guidelines for assessment of bone microstructure in rodents using micro-computed tomography. J. Bone Miner. Res..

[59] Yuan Q (2014). Increased osteopontin contributes to inhibition of bone mineralization in FGF23-deficient mice. J. Bone Miner. Res..

[60] Liu W (2016). GDF11 decreases bone mass by stimulating osteoclastogenesis and inhibiting osteoblast differentiation. Nat. Commun..

[61] Guo Y (2016). Estrogen deficiency leads to further bone loss in the mandible of CKD mice. PLoS ONE.

[62] Zhou CC (2017). AFF1 and AFF4 differentially regulate the osteogenic differentiation of human MSCs. Bone Res..

[63] Guo Yu‐chen, Wang Meng‐yuan, Zhang Shi‐wen, Wu Yun‐shu, Zhou Chen‐chen, Zheng Ri‐xin, Shao Bin, Wang Yuan, Xie Liang, Liu Wei‐qing, Sun Ning‐yuan, Jing Jun‐jun, Ye Ling, Chen Qian‐ming, Yuan Quan (2018). Ubiquitin‐specific protease USP34 controls osteogenic differentiation and bone formation by regulating BMP2 signaling. The EMBO Journal.

[64] Dominissini D, Moshitch-Moshkovitz S, Salmon-Divon M, Amariglio N, Rechavi G (2013). Transcriptome-wide mapping of N(6)-methyladenosine by m(6)A-seq based on immunocapturing and massively parallel sequencing. Nat. Protoc..

[65] Lin S, Choe J, Du P, Triboulet R, Gregory RI (2016). The m(6)A methyltransferase METTL3 promotes translation in human cancer cells. Mol. Cell.

[66] Bo Y (2017). KDM3 epigenetically controls tumorigenic potentials of human colorectal cancer stem cells through Wnt/β-catenin signalling. Nat. Commun..

[67] Yuan Q (2011). FGF‐23/Klotho signaling is not essential for the phosphaturic and anabolic functions of PTH. J. Bone Miner. Res..

