# Supplementary material for: Mettl3-mediated m6A RNA methylation regulates the fate of bone marrow mesenchymal stem cells and osteoporosis
Source: Nat Commun. 2018 Nov 14;9:4772. doi: 10.1038/s41467-018-06898-4 (PMC6235890; doi:10.1038/s41467-018-06898-4)
Supplement: Supplementary file 1 — Supplementary Information [file 41467_2018_6898_MOESM1_ESM.pdf]

# Mettl3-mediated m$^6$A RNA methylation regulates the fate of bone marrow mesenchymal stem cells and osteoporosis

Wu et al.

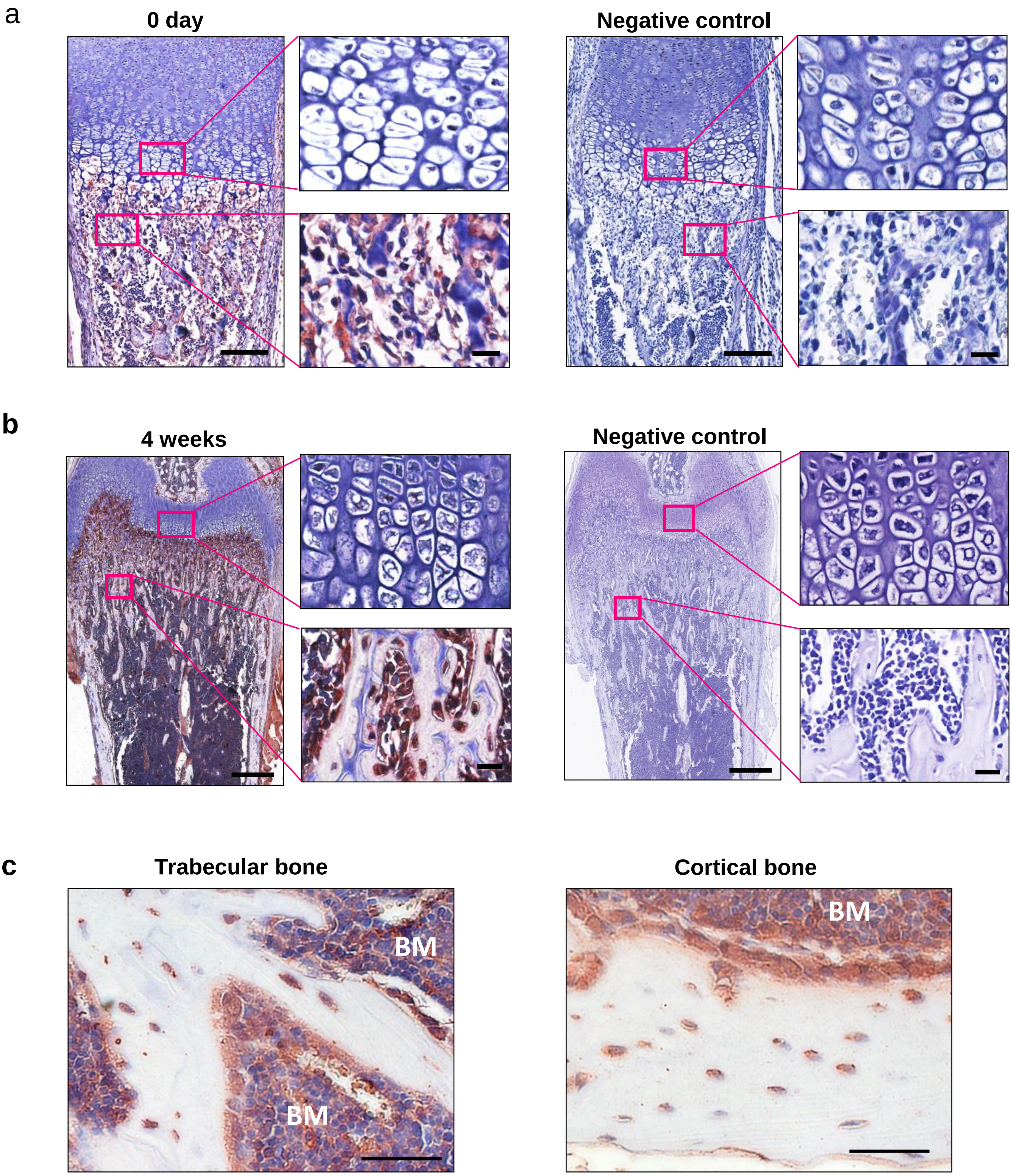


**Supplementary Figure 1 Expression of Mettl3 in trabecular and cortical bone.**
(a-b) Immunohistochemical staining images unraveled that Mettl3 is present in bone marrow and osteoblasts of mice at birth (a) and at 4 weeks of age (b), but is absent in chondrocytes at growth plate. Scale bars, 500μm and 50 μm. (c) Immunohistochemical staining images presenting the expression of Mettl3 in trabecular and cortical bone of 3-month-old mice. BM: bone marrow. Scale bar, 50 μm.

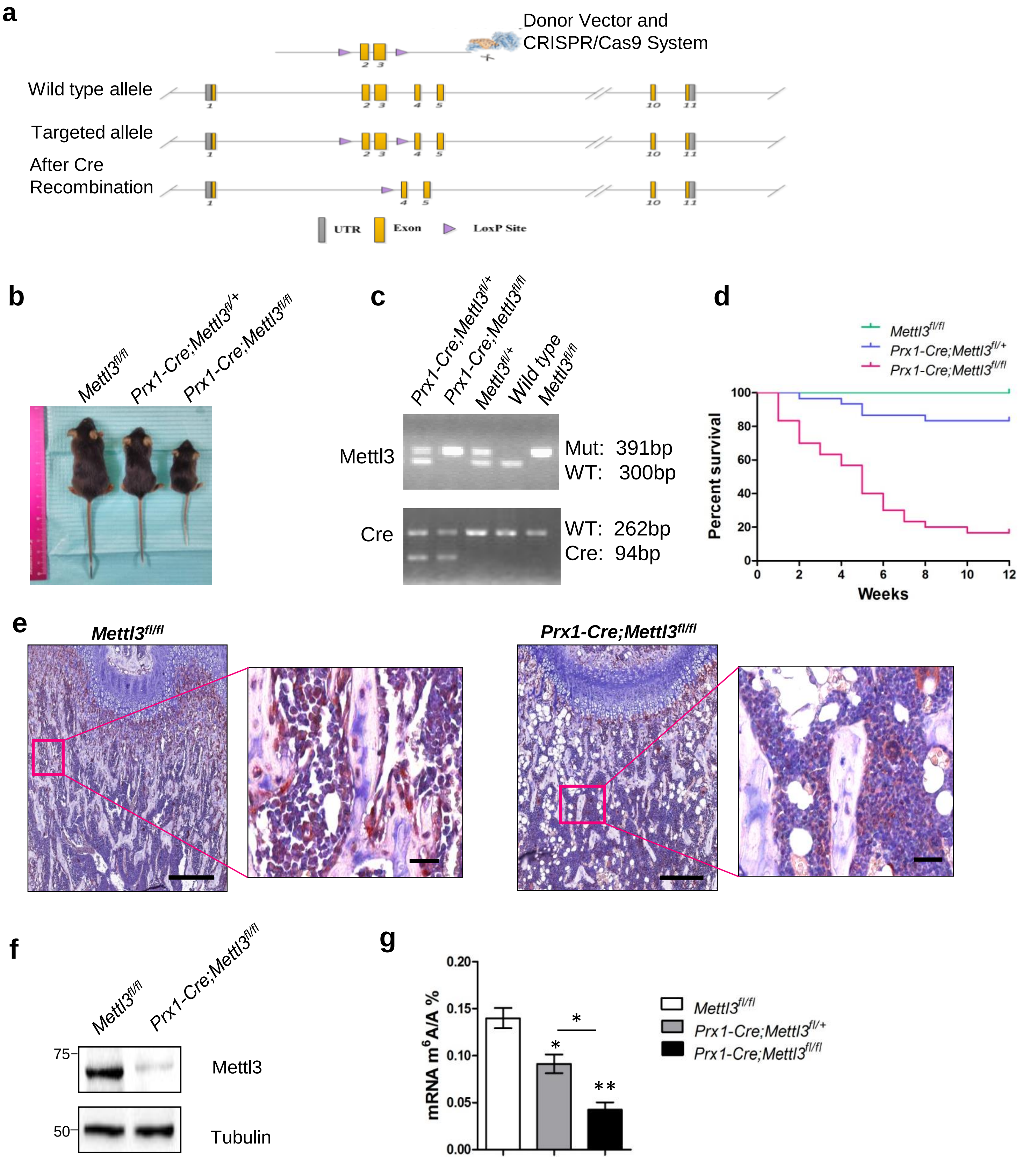


**Supplementary Figure 2 Generation of conditional *Mettl3* knockout mice.**
(a) Schematic representation of conditional *Mettl3* knockout strategy. The exon 2 and exon 3 are deleted after cre-mediated recombination, resulting in translation termination. (b) Representative images of *Mettl3*$^{fl/fl}$, *Prx1-Cre;Mettl3*$^{fl/+}$ and *Prx1-Cre;Mettl3*$^{fl/fl}$ male mice at 4 weeks old. (c) Representative images of PCR genotyping. (d) Survival rate of *Mettl3*$^{fl/fl}$, *Prx1-Cre;Mettl3*$^{fl/+}$ and *Prx1-Cre;Mettl3*$^{fl/fl}$ mice. (e) Immunohistochemical staining confirmed the deletion of *Mettl3* in osteoblasts of *Prx1-Cre;Mettl3*$^{fl/fl}$ mice. Scale bars, 500μm and 50 μm. (f) Western blot analysis of Mettl3. (g) LC-MS/MS analysis of mRNA m$^6$A methylation level in MSCs from *Mettl3*$^{fl/fl}$, *Prx1-Cre;Mettl3*$^{fl/+}$ and *Prx1-Cre;Mettl3*$^{fl/fl}$ mice. Results are from three independent experiments. Data are expressed as mean±s.e.m.; **P*<0.05, ***P*<0.01 by one-way ANOVA with Tukey's *post hoc* test.

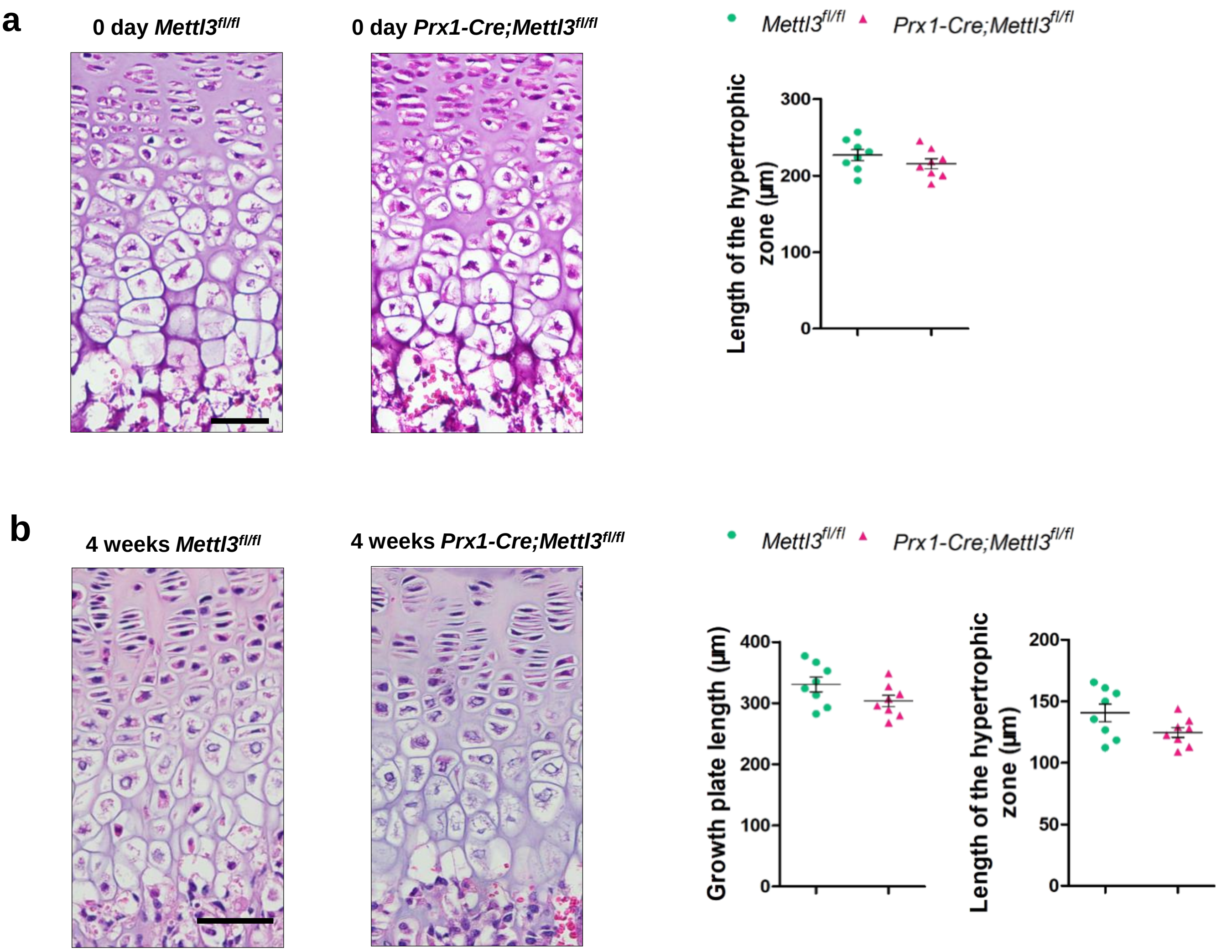


**Supplementary Figure 3 Analysis of growth plate zone in femurs at birth and at 4 weeks old.**

(a) Representing H&E staining images and statistical analysis of growth plate hypertrophic zone. Scale bars, 100μm. *P*=0.273 (b) Representing H&E staining images and statistical analysis of growth plate length (*P*= 0.099) and length of the hypertrophic zone (*P*= 0.073). Scale bars, 100μm. Data are expressed as mean±s.e.m.

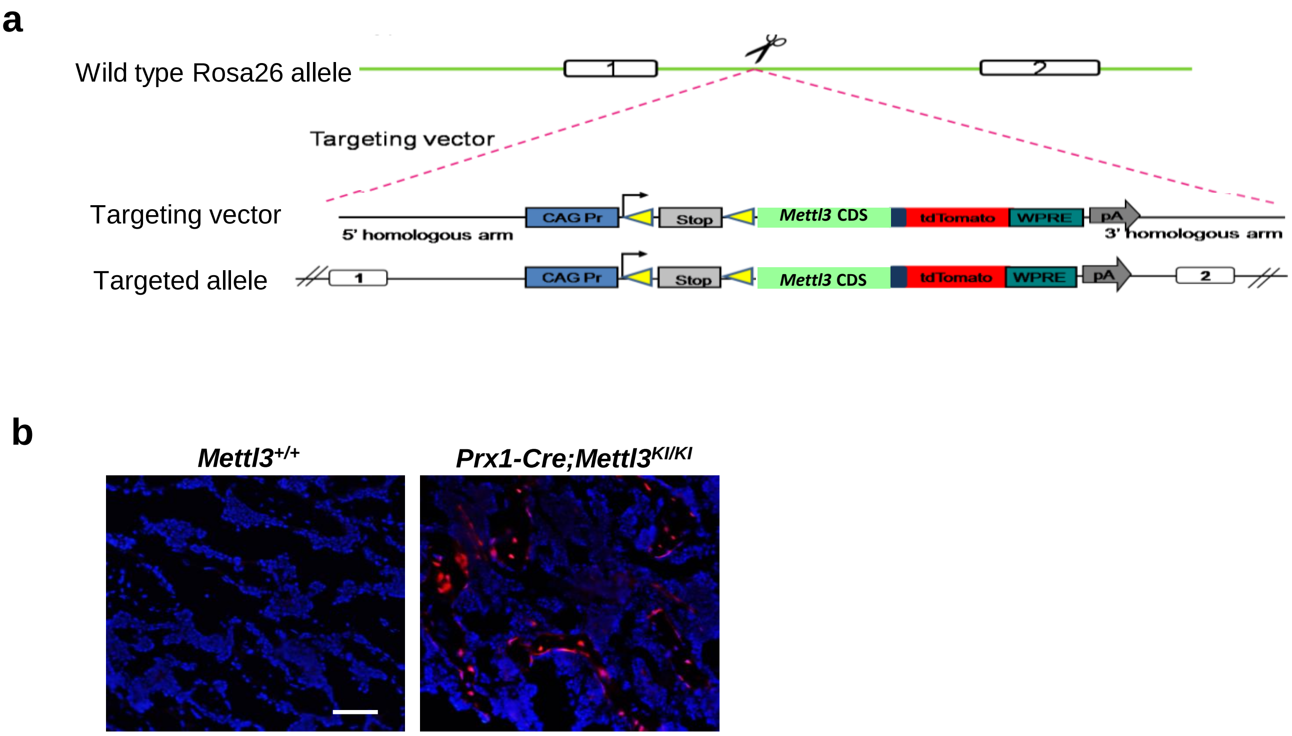


**Supplementary Figure 4 Generation of conditional *Mettl3* knock-in mice.**
(a) Schematic representation of conditional *Mettl3* knock-in strategy. Cre-recombinase will remove the stop codon at the upstream of *Mettl3* coding sequence (CDS), resulting in *Mettl3* over-expression. (b) tdTomato+DAPI merged immunofluoresence images of frozen femur sections from *Prx1-Cre;Mettl3*$^{KI/KI}$ mice and their control littermates, confirming the effective knock-in of *Mettl3* alleles. Scale bar, 50 μm.

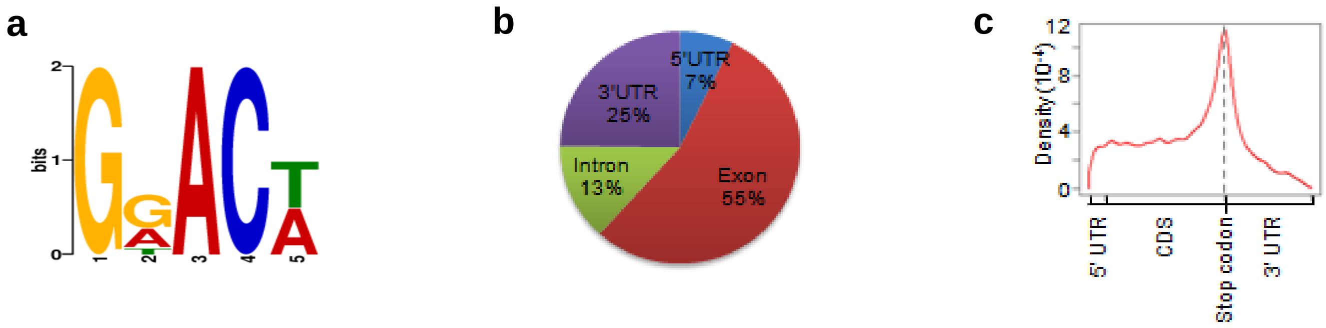


**Supplementary Figure 5 m⁶A features In mouse MSCs.**
(a) The ‘‘GGAC’’ sequence motif identified in MSCs m⁶A peaks. (b) Pie chart representing m⁶A peak distribution. (c) Metagene analyses revealed that the m⁶A sites were predominantly localized near the translation stop codons.

Figure 5f

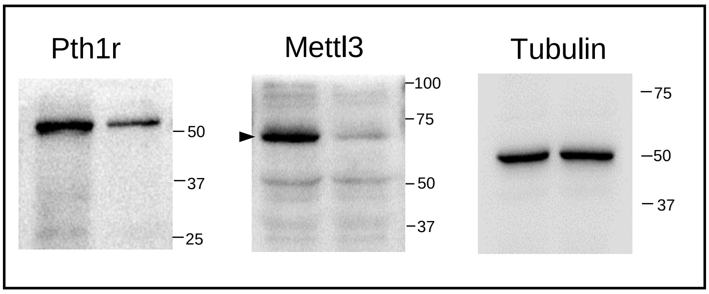


Figure 6b

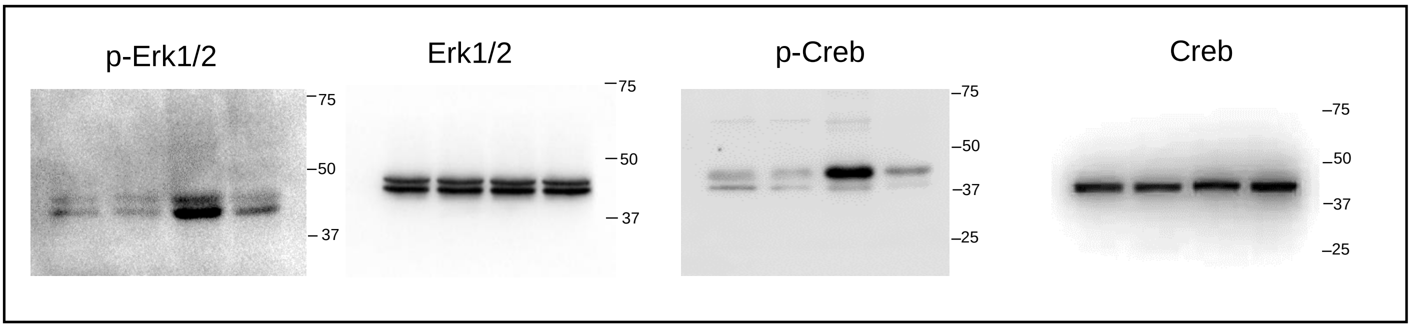


Figure 7a

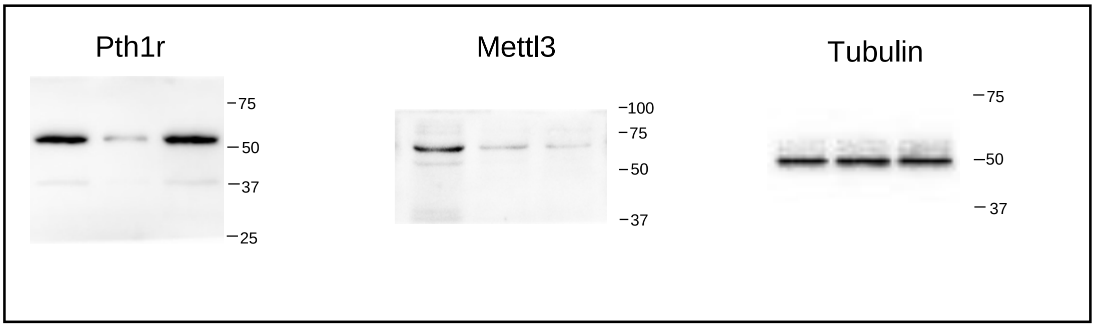


Supplementary Figure 2f

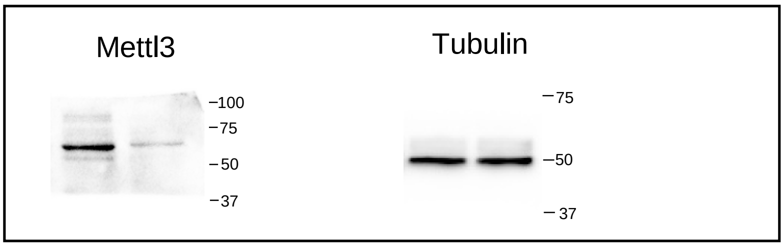


**Supplementary Figure 6 Uncropped images of western blot.**

**Supplementary Table 1. Primers for quantitative RT-PCR**

| | |
|---|---|
| Gapdh F | ACTGAGGACCAGGTTGTC |
| Gapdh R | TGCTGTAGCCGTATTCATTG |
| Runx2 F | GGTACTTCGTCAGCATCCTATCAG |
| Runx2 R | GCTTCCGTCAGCGTCAACAC |
| Sp7 F | ATGGCGTCCTCTCTGCTTG |
| Sp7 R | TGAAAGGTCAGCGTATGGCTT |
| Alp F | AACCCAGACACAAGCATTCC |
| Alp R | GCCTTTGAGGTTTTTGGTCA |
| Bglap F | TTGGTGCACACCTAGCAGAC |
| Bglap R | ACCTTATTGCCCTCCTGCTT |
| 36B4 F | TGAGATTCGGGATATGCTGTTGG |
| 36B4 R | CGGGTCCTAGACCAGTGTTCT |
| C/ebpα F | ACTCCTCCTTTTCCTACCG |
| C/ebpα R | AGGAAGCAGGAATCCTCC |
| Pparg F | CATCAGGCTTCCACTATG |
| Pparg R | CACAGCAAGGCACTTCTG |
| Adiponectin F | CGTCACTGTTCCCAATGT |
| Adiponectin R | ACCGTGATGTGGTAAGAG |
| Plin1 F | CCTGTGGTGAGCGGGACC |
| Plin1 R | GTGGACAGCCGACGGACC |
| CD36 F | GAGCAACTGGTGGATGGTTT |
| CD36 R | GCAGAATCAAGGGAGAGCAC |
| Pth1r F | CAGGCGCAATGTGACAAGC |
| Pth1r R | TTTCCCGGTGCCTTCTCTTTC |
