# Supplementary material for: Mettl3-mediated m6A RNA methylation regulates the fate of bone marrow mesenchymal stem cells and osteoporosis
Source: Nat Commun. 2018 Nov 14;9:4772. doi: 10.1038/s41467-018-06898-4 (PMC6235890; doi:10.1038/s41467-018-06898-4)
Supplement: Supplementary file 3 — Description of Additional Supplementary Files [file 41467_2018_6898_MOESM3_ESM.docx]

**Title:** Supplementary Dataset 1

**Description:** m6 A MeRIP-seq result in MSCs
